# Inflammatory Signal Persistence in Pain: Lymphatic Regulation and Neuroimmune Integration

**DOI:** 10.3390/biology15131024

**Published:** 2026-06-27

**Authors:** Eleonora Solari, Cristiana Marcozzi, Vittorio Vellani, Angela Pignatelli, Andrea Moriondo

**Affiliations:** 1 Department of Medicine and Technological Innovation, University of Insubria, I-21100 Varese, Italy; eleonora.solari@uninsubria.it (E.S.); cristiana.marcozzi@uninsubria.it (C.M.); 2 Department of Neuroscience and Biomedical and Metabolical Sciences, University of Modena and Reggio Emilia, I-41100 Modena, Italy; vvellani@unimore.it; 3 Department of Neurosciences and Rehabilitation, University of Ferrara, I-44121 Ferrara, Italy; pnn@unife.it

**Keywords:** inflammatory pain, neuroimmune integration, CGRP, substance P, lymphatic barrier, lymphatic contractility, lymphangiogenesis

## Abstract

Inflammatory pain arises from the interaction between nociceptive activation and immune signalling within injured or inflamed tissues. While the production of inflammatory mediators has been extensively investigated, less attention has been directed toward mechanisms governing their clearance and spatial redistribution. In this review, we examine how lymphatic vessels actively regulate inflammatory signal persistence in the interstitial space by integrating endothelial barrier behaviour, structural remodelling and intrinsic contractility. The lymphatic system, traditionally regarded as a passive conduit for fluid return and immune cell trafficking, is increasingly recognised as a dynamic regulator of tissue homeostasis. Neuropeptides released from nociceptive afferents, including calcitonin gene-related peptide (CGRP) and substance P (SP), influence lymphatic permeability, lymphangiogenesis and lymph propulsion, thereby shaping the kinetics of inflammatory mediator clearance. Endogenous opioid signalling and pain-related anaesthetic agents may further modulate lymphatic function through effects on lymphatic muscle excitability and endothelial dynamics. Collectively, we propose a systems-level framework in which lymphatic physiology operates as a regulatory interface linking neural activation to immune resolution. Recognising this integrative role may refine current concepts of inflammatory pain and provide a physiologically grounded basis for future translational investigation.

## Simple Summary

Inflammatory pain arises when tissue injury or immune activation leads to the release of chemical signals that sensitise pain nerve endings and prolong discomfort. While much attention has focused on how these signals are produced, less consideration has been given to how they are cleared from tissues. The lymphatic system drains excess fluid, molecules, and transports immune cells. It has traditionally been considered a passive transport route, however, in this review, we discuss evidence showing that lymphatic vessels actively regulate how inflammatory signals spread, persist or resolve within tissues. By controlling fluid entry, vessel growth and rhythmic contractions that propel lymph, lymphatics influence how long inflammatory molecules remain in contact with pain-sensing nerves. Impaired lymphatic function may therefore contribute to the persistence of pain, whereas coordinated lymphatic activity may support recovery. Understanding this regulatory role broadens the current view of inflammatory pain from a purely nerve-centred process to one that also depends on tissue clearance mechanisms. This perspective may help guide future research aimed at improving the management of pain-related inflammatory conditions.

## 1. Introduction

Pain is a fundamental biological response to actual or potential tissue injury and serves an essential protective function by promoting avoidance of harmful stimuli. Under physiological conditions, acute nociceptive pain arises from the activation of specialised nociceptive afferents that detect mechanical, thermal or chemical perturbations and initiate adaptive responses. In contrast, inflammatory and chronic pain states reflect a more complex interplay between peripheral nociceptors, immune mediators and tissue-resident cells, leading to prolonged sensitisation of pain-related pathways and, in some cases, maladaptive amplification of nociceptive signalling [1]. Current understanding of inflammatory pain has therefore increasingly emphasised the neuroimmune interface as a key determinant of nociceptor sensitisation and signal persistence. This perspective integrates the effects of inflammatory mediators, vascular responses and tissue-resident cellular interactions in shaping the interstitial microenvironment surrounding sensitised nociceptors. Moreover, emerging evidence suggests that nutritional factors may modulate this neuroimmune interface by influencing systemic and neural inflammatory responses, thereby affecting pain sensitivity [2,3]. A key feature of inflammatory pain is neurogenic inflammation, whereby nociceptive afferents release bioactive peptides into the surrounding tissue microenvironment. These neuropeptides contribute to vasodilatation, increased vascular permeability, immune cell recruitment and modulation of local tissue responses. Among the best-characterised mediators of this process are calcitonin gene-related peptide (CGRP) and substance P (SP), which are released from peripheral nociceptive terminals in response to tissue stress and inflammatory stimuli. Beyond their direct effects on neuronal excitability, these peptides act on multiple non-neuronal targets within inflamed tissues, shaping the magnitude and persistence of the inflammatory response [4,5]. Although considerable attention has been devoted to the interaction between nociceptors, immune cells and the blood vasculature in the generation of inflammatory pain, the potential contribution of the lymphatic system has remained comparatively underexplored. This relative neglect is striking, given that lymphatic vessels play a central role in regulating interstitial fluid homeostasis, immune cell trafficking and the clearance of inflammatory mediators, all of which directly influence the microenvironment of sensitised nociceptors [6].

Traditionally, the investigation of vascular contributions to inflammatory pain has been largely focused on the blood circulation, with emphasis placed on vasodilatation, plasma extravasation and leukocyte recruitment. In contrast, the lymphatic system has long been regarded as a passive drainage network responsible primarily for returning interstitial fluid, macromolecules and immune cells to the venous circulation [7]. This view has progressively evolved. Accumulating evidence now recognises lymphatic vessels as active regulators of tissue fluid balance, immune surveillance and inflammatory resolution. By controlling the removal of excess IF and the transport of cytokines, antigens and immune cells to regional lymph nodes, lymphatic vessels critically influence the composition and persistence of the inflammatory microenvironment. Impaired lymph drainage and reduced lymph propulsion may lead to tissue oedema, alter immune cell distribution and prolong nociceptor exposure to pro-inflammatory mediators. Conversely, adaptive changes in lymphatic function can facilitate restoration of tissue homeostasis. These properties position the lymphatic network at a strategic interface between the nervous and immune systems, where alterations in fluid dynamics and immune trafficking may substantially affect nociceptive signalling [8]. However, the specific implications of lymphatic physiology for inflammatory signal persistence in pain have remained only partially integrated within current neuroimmune models, particularly across different tissues and disease contexts, where the strength of direct evidence is still lagging.

The close anatomical proximity between nociceptive terminals and lymphatic vessels in many tissues suggests that lymphatics may participate directly in neurogenic inflammatory responses. Nociceptive fibres releasing CGRP and SP are frequently located in perivascular and perilymphatic regions, creating microenvironments in which neural activity can rapidly influence vascular and interstitial dynamics [8,9,10]. In this context, lymphatic vessels should not be considered merely passive recipients of inflammatory by-products, but as potential effectors through which nociceptive signalling can reshape tissue homeostasis. Neuropeptide-dependent modulation of lymphatic function can be conceptualised along three interrelated levels. First, regulation of the lymphatic endothelial barrier influences interstitial fluid uptake and solute clearance, thereby regulating the local concentration of inflammatory mediators. Second, neuropeptides may affect structural remodelling of the lymphatic network through modulation of lymphangiogenesis, altering vessel density and the spatial organisation of immune trafficking. Third, modulation of the intrinsic contractile activity of collecting lymphatics influences lymph propulsion and, consequently, the efficiency of fluid and immune cell transport from peripheral tissues to draining lymph nodes. Together, these levels of regulation provide a framework through which neural signals can influence the magnitude, duration and distribution of inflammatory responses. By shaping barrier function, structural adaptation and contractile behaviour, lymphatic vessels may determine whether inflammatory mediators are rapidly cleared or persist within the interstitium, thereby contributing to the transition from acute adaptive pain to sustained inflammatory or chronic pain conditions [11].

Although much of the early work linking nociceptive neuropeptides and lymphatic function have focused on the meninges in the context of migraine [12], the principles underlying neuropeptide-lymphatic interactions are unlikely to be confined to the central nervous system. Nevertheless, the available evidence remains more constrained in selected settings than across peripheral inflammatory conditions, underscoring the need for a still missing broader integrative physiological framework. Lymphatic vessels are widely distributed throughout peripheral tissues, including skin, gastrointestinal tract, reproductive organs and tumour microenvironments, where they are frequently located in close proximity to nociceptive fibres and immune cell populations [4,12,13]. This anatomical arrangement provides a structural basis for coordinated neuroimmune regulation across diverse organs. In peripheral inflammatory conditions, alterations in lymphatic drainage, vessel density and intrinsic contractility may influence the persistence of oedema, immune cell retention and cytokine gradients within tissues. These changes are of direct relevance to nociceptor sensitisation, as the local inflammatory microenvironment strongly determines activation thresholds and responsiveness of nociceptive afferents. From this perspective, lymphatic dysfunction may represent not only a secondary consequence of inflammation, but also a potential contributor to whether tissue injury resolves or progresses towards sustained inflammatory or chronic pain states. Extending the analysis of CGRP- and SP-mediated effects on lymphatic function beyond a single organ allows the lymphatic network to be considered a systemic modulator of neurogenic inflammation. This broader framework supports the hypothesis that lymphatics contribute to the spatial and temporal organisation of inflammatory signals that shape pain perception across multiple physiological and pathological contexts.

Despite increasing recognition of neuroimmune mechanisms in inflammatory pain, the role of lymphatic physiology in shaping inflammatory signal persistence remains poorly integrated into current conceptual frameworks. To address this gap, we organise current evidence into an integrative framework based on three interconnected levels of lymphatic regulation—endothelial barrier behaviour, structural remodelling and intrinsic contractility—through which nociceptive neuropeptides may influence the resolution or persistence of inflammation across central and peripheral tissues.

## 2. Morphological and Functional Organization of the Lymphatic System

The lymphatic system is organised as a unidirectional vascular network composed of lymphatic capillaries, pre-collecting vessels, lymphatic collectors and lymph nodes, which together ensure the transport of lymph from peripheral tissues back to the blood circulation. This hierarchical organisation enables the coordination of fluid uptake, propulsion and immune cell trafficking across distinct structural segments, each characterised by specific morphological and functional properties [14], thereby determining how efficiently interstitial molecules are removed or redistributed within tissues.

**Figure 1:**
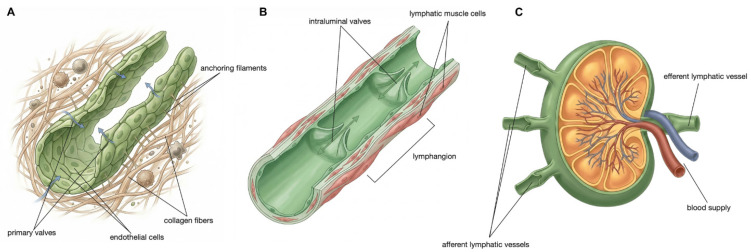
Structural and functional organisation of the lymphatic vasculature. (**A**). Lymphatic capillaries are blind-ended vessels composed of a single layer of LECs connected by discontinuous, button-like intercellular junctions forming primary valves, which allow the entry of interstitial fluid, macromolecules and cells in response to pressure gradients. Lymphatic capillaries are anchored to the surrounding extracellular matrix via anchoring filaments, which transmit mechanical forces and facilitate vessel opening. (**B**). Collecting lymphatics display a more organised structure, with a continuous endothelial lining, intraluminal valves and a surrounding layer of LMCs. Intraluminal valves minimise lymph backflow and define lymphangions, while LMC contractions drive lymph propulsion. (**C**). Lymph nodes are compartmentalised structures receiving lymph via afferent collectors and draining it through efferent vessels. Within the node, lymph flows through specialised sinuses supporting immune interactions and antigen processing. The nodal architecture increases hydraulic resistance and promotes fluid residence time.

Lymphatic capillaries (Figure 1A), also referred to as initial lymphatics, represent the primary sites of lymph formation and drainage and are widely distributed throughout most vascularised tissues. These vessels are blind-ended, thin-walled structures composed of a single layer of lymphatic endothelial cells (LECs). In contrast to blood capillaries, lymphatic capillaries lack a continuous basement membrane and do not possess pericytes, structural features that confer high permeability to interstitial fluid, macromolecules, dietary lipids and cells [15,16]. Adjacent LECs are connected by discontinuous, button-like intercellular junctions that function as primary valves. These specialised junctions allow fluid entry in response to an increase in hydraulic interstitial pressure while limiting retrograde flow into the surrounding interstitium [17,18]. Beyond their role in fluid uptake, these specialised button-like junctions also represent the principal entry route for inflammatory mediators, including cytokines implicated in nociceptor sensitisation (e.g., TNF-α and IL-1β), whose clearance kinetics may influence the duration of nociceptor exposure. Recent evidence suggests that the structural organisation of these junctions is dynamically regulated by the local inflammatory milieu, positioning the lymphatic capillary bed as an early regulatory checkpoint in the control and resolution of neurogenic inflammation [8,12,19,20,21].

Lymphatic capillaries are further anchored to the extracellular matrix through anchoring filaments, which transmit mechanical stresses generated by tissue deformation to the vessel wall, thus promoting luminal opening and facilitating interstitial fluid uptake [22].

From the capillary plexus, lymph is conveyed into pre-collecting vessels and subsequently into lymphatic collectors (Figure 1B), which display a progressively more complex wall organisation. Collecting vessels are characterised by a continuous basement membrane and are surrounded by a lymphatic muscle layer composed of a dense mesh of lymphatic muscle cells (LMCs) arranged in a discontinuous network rather than in the compact concentric organisation typical of arteriolar smooth muscle [23,24,25]. In addition, they display intraluminal secondary valves, which segment the vessel into lymphangions, the lymphatic functional units, and ensure centripetal lymph flow by minimising massive backflow. Lymphangions operate as in-series contractile segments that coordinate lymph propulsion in the absence of a central pump [26,27,28,29]. This organisation is of direct functional relevance, as lymphangion coordination and valve competence determine the efficiency with which fluid, solutes and inflammatory signals are transported from peripheral tissues. Recent studies have further highlighted the importance of lymphangion architecture and valve morphology in maintaining coordinated forward lymph transport, reinforcing the concept that collecting vessel structure is a critical determinant of lymph propulsion and clearance efficiency [27,29]. The structural transition from capillaries to collectors reflects a functional shift from passive fluid entry to active transport. Within collecting vessels, specialised LMCs generate spontaneous, rhythmic contractions of the vessel wall. Although the lymphatic muscle is generally less organised than the smooth muscle layer of arterioles, LMCs density and structural complexity increase along the lymphatic tree, supporting enhanced contractile capacity in more proximal segments. The intrinsic phasic contractions of collecting lymphatics produce cyclical changes in vessel diameter and intraluminal pressure, thereby driving forward lymph propulsion across adjacent lymphangions and, against an overall hydraulic pressure gradient, to the blood venous system [30,31,32,33,34].

Along collecting vessels, lymph nodes (Figure 1C) are strategically positioned and serve both immunological and haemodynamic functions. Structurally encapsulated organs composed of a fibrous capsule and an internal network of lymphatic sinuses lined by specialised LECs [35,36], lymph nodes receive afferent lymphatics that deliver newly formed lymph into the subcapsular sinus. From there, lymph percolates through cortical and medullary sinuses before exiting via efferent vessels at the hilum [36]. The architectural arrangement of nodal sinuses generates regions of relatively low shear stress and prolonged lymph residence time [36], both conditions that favour antigen presentation and immune cell interactions. In addition to their immunological role, lymph nodes impose resistance to flow and therefore contribute to the regulation of upstream lymph propulsion [16,37,38].

Lymph transport relies on the coordinated interplay between intrinsic and extrinsic mechanisms. The intrinsic mechanism is mediated by the spontaneous contractility of the lymphatic muscle, driven by pacemaker activity within specialised LMCs [39,40,41,42,43,44,45]. This contractile behaviour is sensitive to changes in transmural pressure, wall stretch, shear stress generated by lymph flow, and local changes in the surrounding microenvironment, and can be modulated by the autonomic nervous system [46,47,48,49,50,51,52]. Endothelial-derived mediators, including nitric oxide (NO), as well as prostaglandins and inflammatory signals, modulate LMC excitability and contraction frequency, thereby adjusting lymph propulsion to local physiological demands [53,54,55,56,57]. Recent evidence [55] has further shown that flow-dependent regulation of lymphatic contractility depends on active endothelial sensing mechanisms, including TRPV4-dependent pathways. This reinforces the view that lymphatic contractility is a dynamically regulated process that adapts to local mechanical and intraluminal conditions through coordinated endothelial–LMC coupling. These regulatory mechanisms are particularly relevant under inflammatory conditions, in which changes in lymphatic pump efficiency may influence the residence time of inflammatory mediators within the interstitium. Extrinsic mechanisms depend on mechanical stresses applied to lymphatics by surrounding tissues. Skeletal muscle contraction, vasomotion, respiratory movements, and tissue displacement intermittently compress and expand lymphatic segments, generating pressure gradients that favour lymph drainage and forward transport [58,59,60,61,62]. The effectiveness of extrinsic forces is determined by vessel compliance and valve competence, which together ensure centripetal lymph flow. Intrinsic and extrinsic mechanisms operate in concert: extrinsic forces dominate in regions exposed to pronounced mechanical stresses and/or undergoing cyclic movements, such as skeletal muscles and the thoracic cavity, whereas intrinsic contractility plays a pivotal role in tissues characterised by limited external motion, including mesenteric and cutaneous regions. If extrinsic flow is adequate to the tissue needs, intrinsic lymph flow adapts and spontaneous contractility is reduced [39,47,63]. Regional variability in lymphatic structure and function reflects the mechanical and physiological characteristics of the surrounding tissue [64,65]. In soft tissues with low baseline mechanical stress, intrinsic phasic contractions are essential for maintaining basal lymph propulsion and interstitial fluid homeostasis. In contrast, in highly moving tissues, extrinsic forces substantially contribute to lymph transport. Disruption of either contractile behaviour, valve competence or structural continuity of lymphangions can markedly impair lymph flow, leading to fluid accumulation, altered immune cell distribution and changes in the interstitial microenvironment. Such regional variability is likely to influence the efficiency with which distinct lymphatic beds clear fluid and inflammatory mediators. Consequently, tissue-specific differences in lymphatic organisation and transport mechanisms may contribute to regional variation in the persistence and resolution of inflammatory signals across organs [65]. Within peripheral tissues, lymphatic capillaries and collecting vessels are embedded in a three-dimensional extracellular matrix that also contains blood vessels, immune cells and nerve fibres. The close spatial arrangement between lymphatics and perivascular neural structures provides an anatomical substrate through which local mechanical forces and cellular interactions may be integrated at the tissue level. This structural organisation establishes the lymphatic system as a dynamic component of tissue fluid regulation and immune surveillance across diverse organs and provides the physiological basis by which altered fluid clearance and lymph propulsion may influence the inflammatory milieu surrounding sensitised nociceptors.

## 3. Regulation of Lymphatic Function by Nociceptive Neuropeptides

### 3.1. Modulation of Lymphatic Barrier Function and Fluid Clearance

**Figure 2:**
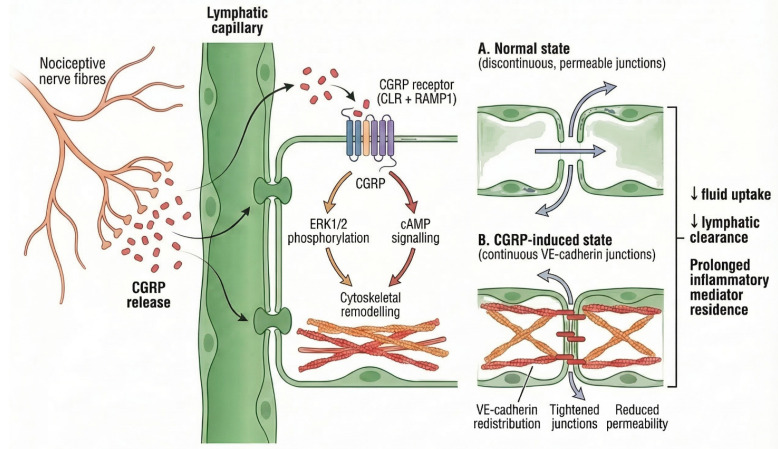
CGRP-mediated modulation of lymphatic endothelial barrier function and fluid clearance. Nociceptive fibres release CGRP, which acts on LECs through the receptor complex of CLR and RAMP1. Receptor activation triggers intracellular signalling pathways, including ERK1/2 phosphorylation and cAMP-dependent signalling, leading to cytoskeletal remodelling and VE-cadherin redistribution at intercellular junctions. (**A**). Under normal conditions, LECs junctions are discontinuous and permeable, allowing efficient entry of interstitial fluid. (**B**). In contrast, CGRP-induced signalling promotes junction tightening, resulting in reduced endothelial permeability. This barrier reinforcement limits fluid uptake into lymphatic capillaries, leading to decreased interstitial fluid clearance and prolonged residence of inflammatory mediators within the tissue microenvironment.

The lymphatic endothelium represents a dynamically regulated barrier that governs lymph entry from the interstitium and serosal cavities. Unlike blood vascular endothelium, LECs are organised in discontinuous junctional structures that allow pressure-dependent entry of fluid (Figure 1A) while preserving unidirectional flow [21]. The integrity and spatial organisation of intercellular junctions are therefore critical determinants of tissue fluid homeostasis and solute clearance, both under physiological and inflammatory conditions (Figure 2A) [18,66]. Emerging evidence indicates that CGRP released from nociceptive afferents directly modulates lymphatic endothelial barrier properties, thereby influencing the efficiency of interstitial fluid drainage and immune trafficking. LECs express functional components of the CGRP receptor complex, including the calcitonin receptor-like receptor (CLR) and receptor activity-modifying protein 1 (RAMP1), which together confer specificity for CGRP binding [12]. Activation of this receptor complex initiates intracellular signalling cascades that include phosphorylation of p44/42 mitogen-activated protein kinase (MAPK/ERK1/2) and modulation of cyclic adenosine monophosphate (cAMP)-dependent pathways [19,67]. In cultured dermal and meningeal LECs, CGRP exposure induces rapid cytoskeletal rearrangement and redistribution of junctional proteins, particularly vascular endothelial cadherin (VE-cadherin), a key regulator of endothelial adhesion and permeability [12,19]. CGRP signalling promotes reorganisation of VE-cadherin at intercellular contacts, leading to altered junctional continuity and modifications in barrier properties. Experimental observations in isolated lymphatic preparations and in vivo models indicate that CGRP reduces lymphatic endothelial permeability and limits fluid uptake under certain inflammatory conditions [12]. These recent studies have further clarified that these effects are closely associated with changes in junctional organisation, rather than with nonspecific endothelial activation alone. This modulation appears to depend on receptor-specific activation, as pharmacological blockade of CLR/RAMP1 or genetic deletion of receptor components in LECs abrogates these effects. The resulting tightening of lymphatic endothelial junctions may reduce the efficiency of cerebrospinal fluid (CSF) or interstitial fluid entry into lymphatic capillaries, thereby altering downstream clearance kinetics [12].

The most compelling evidence for CGRP-mediated modulation of lymphatic barrier function derives from studies of meningeal lymphatic vessels in experimental models of migraine [12]. Accordingly, the current evidence base is strongest in meningeal settings, whereas the relevance of these mechanisms to peripheral inflammatory pain remains less directly established. In these models, selective deletion of CGRP receptor components in LECs attenuates photophobia and pain-related behaviours [68], suggesting that lymphatic CGRP signalling contributes to neuroinflammatory amplification. At the tissue level, CGRP administration is associated with redistribution of VE-cadherin within meningeal lymphatic endothelium [12] and reduced fluid uptake into draining cervical lymph nodes, consistent with impaired CSF clearance (Figure 2B). In cultured human LECs, an elevated level of CGRP was found to induce formation of continuous, nonpermeable VE-cadherin junctions [19], involving activation of ERK-CREB phosphorylation [12,19]. Thus, CGRP signaling was required for VE-cadherin linear junction formation at meningeal lymphatic endpoints to render the barrier impermeable and to impair the drainage function. Complementary studies using dynamic contrast-enhanced MRI demonstrate delayed meningeal lymphatic wash-in and reduced transfer constants in patients with episodic migraine, findings that correlate with headache severity [20,69,70]. Although recent imaging approaches do not directly quantify endothelial junctional rearrangement, they provide functional support for the role of lymphatic barrier function in determining clearance dynamics in vivo [20], thus strengthening the translational relevance of the meningeal findings, while also highlighting the need for comparable functional studies in peripheral tissues.

Beyond the meninges, CGRP-dependent regulation of lymphatic endothelial signalling has been observed in peripheral tissues [71]. In dermal LECs, CGRP induces time- and dose-dependent activation of ERK1/2 and alters cytoskeletal organisation, suggesting that barrier modulation is not restricted to a single anatomical compartment [19,72]. In peripheral tissues, the available evidence more directly supports endothelial responsiveness, whereas its contribution to pain-related inflammatory persistence remains less clearly defined. However, the observed effects on endothelial junctional organisation suggest that CGRP-dependent barrier regulation may influence the balance between inflammatory mediator retention and lymphatic uptake. Given that lymphatic capillaries are embedded within an extracellular matrix rich in immune cells and inflammatory mediators, even modest changes in junctional architecture may substantially influence the local interstitial microenvironment. Reduced interstitial fluid and solute uptake can prolong the residence time of cytokines, chemokines and other bioactive molecules, thereby modifying immune cell activation and spatial distribution within tissues [38].

Although SP has been more prominently associated with vascular permeability in blood vessels, evidence suggests that neurokinin-1 receptor (NK1R) signalling may also influence lymphatic endothelial behaviour. NK1R expression has been reported in LECs, and SP exposure can modulate endothelial cytoskeletal dynamics and intracellular Ca^2+^ signalling [69,73,74,75]. However, current data indicate that SP exerts a comparatively more robust influence on lymphangiogenesis and immune-mediated remodelling rather than on acute barrier tightening [76,77].

Therefore, CGRP appears to represent a major nociceptive neuropeptide regulator of LECs junctional organisation and fluid clearance [67]. These findings establish the lymphatic endothelium as a neuropeptide-sensitive interface through which neural activity can regulate interstitial fluid handling. By modulating LECs junctional architecture and permeability, CGRP can alter the efficiency of solute uptake and downstream lymph transport, thereby influencing the composition and persistence of the local inflammatory microenvironment, thus providing a molecular framework linking nociceptive afferent activity to changes in lymphatic clearance across central and peripheral tissues.

### 3.2. Neuropeptide-Driven Lymphangiogenesis and Structural Remodelling

**Figure 3:**
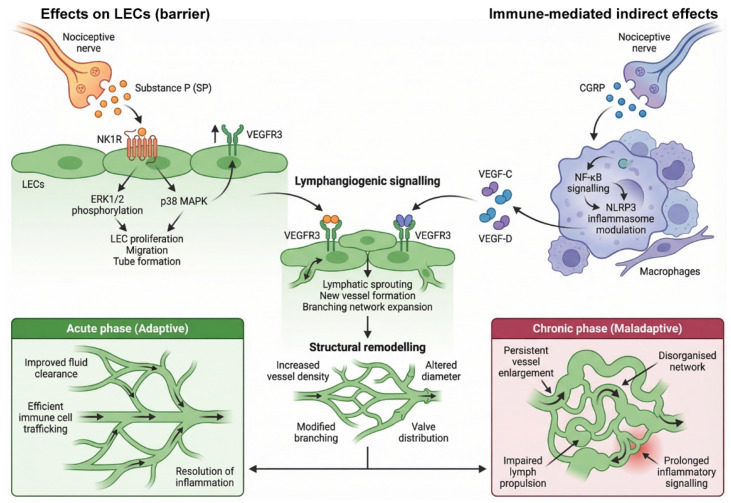
Neuropeptide-driven lymphangiogenesis and structural remodelling of lymphatic vessels. Nociceptive neuropeptides regulate lymphatic growth through direct endothelial and indirect immune-mediated mechanisms. SP released from nociceptive fibres directly stimulates LECs via NK1R, promoting proliferation, migration and tube formation, and upregulating VEGFR3 expression. In parallel, CGRP acts through immune intermediates, including macrophages, modulating NF-κB signalling and NLRP3 inflammasome activity, leading to increased production of VEGF-C and VEGF-D. These ligands converge on VEGFR3 signalling in LECs, driving lymphatic sprouting, new vessel formation and network expansion. Structural remodelling includes increased vessel density, altered diameter, branching patterns and valve distribution. Lymphangiogenesis exhibits a context-dependent dual role: during acute inflammation it supports fluid clearance, immune cell trafficking and resolution of inflammation, whereas prolonged neuropeptide signalling promotes persistent vessel enlargement, network disorganisation, impaired lymph propulsion and sustained inflammatory signalling within the tissue microenvironment.

Lymphangiogenesis is the formation of new lymphatic vessels from pre-existing lymphatic structures and represents a highly plastic component of the inflammatory response. Under physiological conditions, lymphatic expansion contributes to tissue repair by facilitating interstitial fluid clearance, restoring immune cell trafficking and re-establishing tissue fluid homeostasis [16,78]. However, under persistent inflammatory conditions, structural remodelling of the lymphatic network may become maladaptive, altering vessel density, architecture and functional efficiency in ways that can stabilise the inflammatory microenvironment. Within this context, nociceptive neuropeptides emerge as important regulators of lymphatic growth and structural adaptation. Among these mediators, SP has been most consistently associated with lymphangiogenic signalling (Figure 3). SP is released from activated nociceptive afferents during tissue injury and inflammation, and exerts its effects primarily through the NK1R receptor, which is expressed by immune and stromal cells, as well as by LECs, in several tissues [76]. In vitro studies using primary human LECs demonstrate that SP increases the expression of vascular endothelial growth factor receptor 3 (VEGFR3), the principal receptor mediating lymphatic growth responses [76]. Activation of NK1R in LECs promotes proliferation and tube formation and is associated with increased VEGFR3 expression [69]. In parallel, SP signalling has been shown to activate ERK1/2 and p38 MAPK pathways in endothelial cells in a NK1R-dependent manner, supporting pro-angiogenic responses [79]. These effects are abolished by NK1R antagonism or receptor silencing, indicating receptor specificity and supporting a direct pro-lymphangiogenic role for SP at the endothelial level [76].

Beyond direct endothelial stimulation, SP also drives lymphatic remodelling indirectly through immune modulation (Figure 3). In inflamed tissues, macrophages exposed to SP undergo phenotypic shifts that enhance the production of canonical lymphangiogenic growth factors, including vascular endothelial growth factors C and D (VEGF-C and VEGF-D) [77]. These ligands stimulate VEGFR3 signalling in neighbouring LECs, amplifying vessel sprouting and network expansion. SP-dependent macrophage polarisation is associated with modulation of NF-KB signalling and regulation of inflammasome-related pathways, including NLRP3 activity, thereby linking neuropeptide release to immune-driven vascular remodelling [77]. In experimental models of tissue injury, exogenous SP administration promotes lymphatic regeneration and reduces interstitial fluid accumulation, effects that correlate with increased LECs proliferation and improved overall vessel organisation [80].

The structural consequences of SP-driven lymphangiogenesis extend beyond simple increases in vessel number. Newly formed lymphatic segments often display altered diameter, branching patterns and valve distribution [81], potentially influencing interstitial fluid uptake, lymph propulsion and solute transport.

In early inflammatory phases, such expansion may enhance interstitial fluid drainage and accelerate removal of inflammatory mediators, thereby contributing to resolution. However, prolonged or excessive SP signalling can promote persistent lymphatic enlargement and aberrant network architecture, particularly within chronically inflamed tissues. Under such conditions, increased vessel density may facilitate continued immune cell trafficking and antigen presentation, thereby reinforcing inflammatory loops within the local microenvironment. Thus, SP-dependent lymphangiogenesis exhibits a dual profile: reparative during acute injury, while potentially stabilising chronic inflammatory niches when neuropeptide release becomes prolonged.

In contrast to the more direct endothelial actions of SP, CGRP appears to regulate lymphangiogenesis predominantly through indirect neuroimmune pathways [71]. Although LECs express components of the CGRP receptor complex in several tissues, robust proliferative responses are more frequently observed in immune and stromal intermediates. Macrophages and fibroblasts exposed to CGRP increase transcription of VEGF-C and VEGF-D, thereby promoting secondary activation of VEGFR3-dependent signalling in adjacent LECs. Genetic ablation or pharmacological inhibition of receptor components, such as RAMP1 in immune cells, reduces inflammation-associated lymphatic expansion in peripheral tissues [82], supporting the concept that CGRP-dependent lymphangiogenesis is largely mediated by modulation of the surrounding cellular milieu rather than direct endothelial proliferation [19]. The distinction between SP and CGRP highlights tissue-specific variability in nociceptive neuropeptide-lymphatic interactions. In highly innervated inflammatory environments enriched in immune infiltrates, CGRP may act primarily as an immunomodulatory signal that shapes the cytokine and growth factor landscape, indirectly determining the extent of lymphatic remodelling. By contrast, SP more readily engages endothelial NK1R-dependent pathways, exerting direct structural effects on the lymphatic network. Nevertheless, both neuropeptides ultimately converge on the VEGFR3-dependent growth pathways, underscoring the central role of canonical lymphangiogenic signalling in neurogenic vascular adaptation [71,76].

Structural remodelling of lymphatics also influences the mechanical properties of the surrounding interstitial tissue. Increased lymphatic density and altered vessel diameter may modify interstitial hydraulic resistance and interstitial fluid distribution. These biomechanical changes can affect the extracellular matrix organisation and local tissue compliance, thereby indirectly influencing immune cell migration and the persistence of inflammatory mediators. In addition, expanded lymphatic networks provide additional conduits for immune cell egress to draining lymph nodes, potentially amplifying adaptive immune responses. Consequently, neuropeptide-driven lymphangiogenesis does not simply alter vessel number but reshapes the spatial organisation of immune communication within tissues. The functional implications of these structural changes are strongly context dependent. Coordinated lymphatic expansion may be adaptive during acute inflammation, whereas persistent or disorganised remodelling may become maladaptive under chronic inflammatory conditions. Indeed, during acute inflammation, organised lymphangiogenesis may represent an adaptive response that restores efficient fluid clearance and limits interstitial fluid accumulation [83]. Enhanced VEGF-C/VEGF-D signalling can promote organised vessel sprouting, improved valve formation and re-establishment of effective lymph propulsion [81]. However, when nociceptive neuropeptide release becomes chronic, prolonged growth factor production may generate disorganised or hyperplastic networks (Figure 3) with suboptimal barrier and contractile function [83]. Such maladaptive remodelling may compromise coordinated lymph drainage and propulsion despite increased vessel density, thereby maintaining interstitial fluid retention and prolonging exposure of resident cells to inflammatory mediators [84]. Thus, neuropeptide-driven lymphangiogenesis should be viewed as a dynamic structural adaptation positioned at the interface between neural activation and immune regulation. SP predominantly acts as a direct pro-lymphangiogenic mediator engaging NK1R-dependent signalling in LECs and immune cells, whereas CGRP more commonly reshapes lymphatic growth indirectly through modulation of the inflammatory microenvironment. In both cases, the resulting structural remodelling alters the architecture of lymphatic networks and the spatial distribution of immune trafficking pathways within the interstitium. The balance between reparative expansion and maladaptive persistence likely determines whether lymphatic remodelling contributes to resolution of inflammation or to stabilisation of chronic inflammatory states.

### 3.3. Modulation of Spontaneous Lymphatic Contractility by Neuropeptides

As stated above (Section 2), spontaneous contractility of lymphatic collectors represents a fundamental determinant of lymph propulsion and lymph transport from peripheral tissues to draining lymph nodes. Unlike lymphatic capillaries, which primarily regulate fluid entry, collecting vessels generate intrinsic, rhythmic contractions through the coordinated activity of LMCs organised within the lymphatic muscle (Figure 1B). These phasic contractions segmentally compress lymphangions, producing cyclical increases in intraluminal pressure that drive forward lymph propulsion in the absence of a central pump. The efficiency of this intrinsic mechanism depends on tightly regulated interactions between LMCs excitability, endothelial signalling and valve competence, and is highly sensitive to changes in the surrounding microenvironment, including inflammatory mediators and neural inputs [33,85].

**Figure 4:**
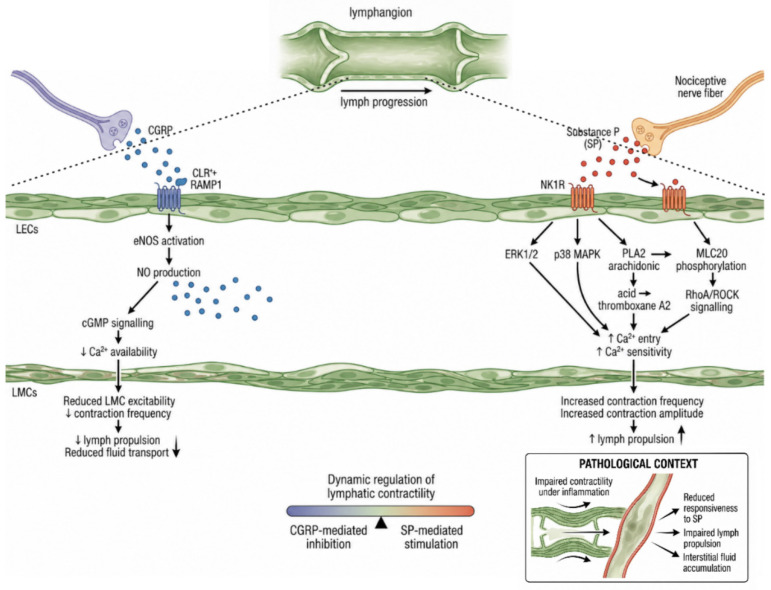
Neuropeptide-dependent modulation of spontaneous lymphatic contractility and lymph propulsion. Collecting vessels generate intrinsic rhythmic contractions through coordinated activity of LMCs organised around lymphangions, enabling active lymph propulsion in the absence of a central pump. This contractility is dynamically regulated by nociceptive neuropeptides through distinct mechanisms. CGRP released from nociceptive afferents acts primarily on LECs via the CLR/RAMP1 receptor complex, inducing eNOS activation and NO production. NO diffuses to adjacent LMCs, where it activates cGMP-dependent signalling, reduces Ca^2+^ availability and suppresses excitability, resulting in decreased contraction frequency and reduced lymph propulsion. In contrast, SP exerts a facilitatory effect through NK1R expressed in LECs and LMCs. SP signalling engages ERK1/2 and p38 MAPK pathways and phospholipase A2-mediated production of arachidonic acid metabolites, such as thromboxane A2, leading to increased Ca^2+^ entry, enhanced Ca^2+^ sensitivity and MLC20 phosphorylation via RhoA/ROCK-dependent mechanisms. These pathways increase contraction frequency and amplitude, enhancing lymph propulsion. The balance between CGRP-mediated inhibition and SP-mediated stimulation determines overall contractile behaviour and lymph transport efficiency. Under pathological conditions, including sustained inflammation, reduced responsiveness to SP may impair contractility, leading to diminished lymph propulsion and interstitial fluid accumulation.

CGRP exerts a predominantly inhibitory influence on spontaneous lymphatic contractility. LECs express functional components of the CGRP receptor complex, including CLR and RAMP1, enabling direct endothelial responsiveness to neuropeptide release from nociceptive afferents. Receptor activation stimulates intracellular signalling pathways that increase NO production via endothelial nitric oxide synthase (eNOS). NO diffuses from LECs to adjacent LMCs, where it activates soluble guanylate cyclase, elevates cGMP levels and reduces cytosolic Ca^2+^ availability (Figure 4). This signalling cascade reduces LMCs excitability and, in turn, suppresses the frequency of spontaneous contractions [85,86]. Recent experimental studies in isolated mesenteric lymphatics have demonstrated that NO reduces contraction frequency and lymphatic pumping through endothelium-dependent mechanisms [53,54], suggesting that CGRP-induced NO production may underlie its inhibitory effects on lymphatic contractility. These findings also support an endothelial-mediated control of lymphatic contractility that is dynamically coupled to local flow and pressure-dependent conditions (Figure 4). Pharmacological inhibition of NO synthase or blockade of CGRP receptor components abolishes this response, supporting the specificity of the CLR/RAMP1-eNOS-NO axis. In addition to reducing Ca^2+^ influx through voltage-dependent Ca^2+^ channels, NO-mediated signalling may decrease Ca^2+^ sensitivity of the contractile apparatus by modulating myosin light chain phosphorylation via cGMP-dependent protein kinase pathways. The resulting net effect is a reduction in chronotropic activity and diminished lymph propulsion, without necessarily abolishing basal tone [87]. Functionally, this CGRP-dependent suppression of contractility complements the barrier-tightening effects. While junctional remodelling limits fluid entry into lymphatic capillaries, inhibition of collecting vessel contractility limits downstream transport of lymph. Together, these mechanisms can substantially reduce interstitial fluid clearance under inflammatory conditions characterised by sustained nociceptive neuropeptide release. From a physiological perspective, transient CGRP-mediated suppression of contractility may represent an adaptive response, redistributing fluid dynamics during acute tissue stress. However, prolonged elevation of CGRP levels, as observed in chronic inflammatory states, may stabilise reduced lymph propulsion and contribute to interstitial fluid retention within the interstitial microenvironment.

In contrast to CGRP, SP displays a more complex and often facilitatory influence on spontaneous lymphatic contractility [70]. NK1R expression has been reported in both LECs and LMCs, allowing SP to act through multiple cellular compartments within the lymphatic wall [69,74] (Figure 4). In isolated collecting lymphatics, SP increases contraction frequency and may also enhance contraction amplitude [69]. These positive chronotropic and inotropic effects appear to involve NK1R coupling to pertussis toxin -sensitive G proteins, leading to activation of phospholipase A2 and subsequent production of arachidonic acid-derived metabolites, including thromboxane A2, which acts as a diffusible mediator that increases LMCs excitability and, in turn, promotes contractile activity and increases contraction frequency [69,86]. Moreover, SP signalling has been shown to activate ERK1/2 and p38 MAPK pathways in a NK1R-dependent manner supporting pro-contractile responses [69]. These pathways may enhance Ca^2+^ entry and increase in Ca^2+^ sensitivity of the actomyosin apparatus, potentially via RhoA/ROCK-dependent modulation of myosin light chain phosphatase activity [88] (Figure 4). The overall result is an increase in contraction frequency and improved lymph propulsion under certain physiological conditions [74].

The endothelial compartment may further modulate SP effects, as lymphatic endothelium releases vasoactive mediators that regulate contractile activity and coordination of lymphatic pumping [89,90]. The balance between contractile signalling and endothelial-derived relaxing factors, including NO and prostaglandins, likely determines the net outcome [53,54]. Consequently, SP effects on contractility may vary according to tissue type, inflammatory state and receptor density. Under pathological conditions, lymphatic responsiveness to SP can be impaired. In models of systemic inflammation and haemorrhagic shock, collecting lymphatics exhibit reduced contractile activity and diminished sensitivity to SP [91]. This uncoupling between neuropeptide signalling and vessel contractility is associated with decreased lymph propulsion and increased interstitial fluid accumulation into the interstitium, suggesting that intact SP signalling contributes to physiological neurogenic regulation of lymphatic contractions, whereas inflammatory dysregulation may compromise this adaptive mechanism [84].

The modulation of spontaneous contractility by nociceptive neuropeptides therefore represents a rapid and functionally reversible level of lymphatic control, distinct from the slower structural adaptations described above. By altering contraction frequency and amplitude, as well as coordination across lymphangions, CGRP and SP can dynamically regulate lymph transport and immune cell egress. Changes in lymph propulsion efficiency directly influence the residence time of cytokines, chemokines and other bioactive mediators within the interstitium, thereby shaping the inflammatory microenvironment experienced by nociceptors. In this perspective, spontaneous lymphatic contractility serves as a functional interface between neural activation and tissue fluid homeostasis. CGRP-mediated suppression of LMC activity tends to reduce lymph propulsion and may stabilise pro-inflammatory conditions when sustained. SP-mediated enhancement of contractility may facilitate interstitial fluid clearance during early inflammatory phases, while prolonged or dysregulated signalling may contribute to maladaptive alterations in propulsion dynamics. The context-dependent balance between these opposing influences likely determines whether neuropeptide release supports resolution of inflammation or promotes persistence of inflammatory pain states. These mechanisms establish lymphatic intrinsic contractility as a neuropeptide-sensitive physiological process through which nociceptive afferents can rapidly influence interstitial fluid handling, immune trafficking and the spatial organisation of inflammatory signals. In this framework, collecting lymphatics operate not merely as passive conduits for fluid transport but as dynamic effectors translating neural signals into coordinated changes in lymph propulsion across central and peripheral tissues [12].

## 4. Neuroimmune Coupling via Lymphatic Vessels in Pain-Related Diseases

Lymphatic vessels are strategically positioned at the interface between nociceptive afferents and immune cell networks, where they regulate both the physical removal of interstitial fluid and the spatial organisation of inflammatory signalling [11,12,14]. Through their control of barrier properties, structural remodelling and intrinsic contractility (see Section 3), lymphatics influence the magnitude of inflammatory responses and spatio-temporal distribution. In this context, lymphatic vessels emerge as dynamic components of neuroimmune coupling. Neurogenic inflammation induced by prolonged activation of nociceptive afferents leads to the release of CGRP and SP within the local microenvironment, which act on immune, stromal and vascular elements [4]. By modulating the three interconnected levels of lymphatic function previously described, nociceptive neuropeptides can alter the efficiency of interstitial fluid clearance and immune cell trafficking, thereby influencing mediator residence time within tissues and the persistence of nociceptor sensitisation [11]. Across pain-related conditions, the degree of direct experimental support for this framework remains variable, particularly when extending mechanistic interpretations from meningeal models to peripheral inflammatory tissues.

The meningeal compartment provides a paradigmatic example of this integration. Meningeal lymphatics regulate CSF outflow and facilitate the transport of immune mediators towards deep cervical lymph nodes [12]. In experimental models characterised by elevated CGRP signalling, alterations in meningeal lymphatic drainage are associated with delayed clearance of inflammatory mediators and enhanced pain-related behaviours [12,20]. Conversely, selective modulation of lymphatic responsiveness to CGRP attenuates neuroinflammatory amplification. Functional imaging studies in patients with migraine further suggest that altered meningeal lymphatic dynamics correlate with symptom severity, supporting the hypothesis that impaired lymphatic clearance contributes to prolonged nociceptive signalling [20,92]. Together, experimental and imaging evidence currently identifies the meningeal compartment as the best-supported example of neuroimmune–lymphatic integration in pain-related conditions. In this framework, lymphatics regulate central neuroimmune communication by controlling the export kinetics of inflammatory signals from the meningeal compartment.

Similar principles apply to peripheral tissues, where lymphatic vessels are embedded within a three-dimensional extracellular matrix that contains nociceptive fibres, immune cells and blood vessels. Although these mechanisms provide a useful conceptual basis, they cannot be directly extrapolated to peripheral tissues without tissue-specific validation, as lymphatic organisation, immune composition and mechanical environment differ substantially across organs. Nevertheless, the available evidence supports the broader relevance of lymphatic regulation in shaping local inflammatory signal handling in several peripheral tissues. For example, in cutaneous inflammatory conditions, reduced lymph flow or maladaptive lymphangiogenesis may prolong interstitial fluid retention and increase local concentrations of cytokines and growth factors that directly lower nociceptor activation thresholds, thereby promoting sensitisation [78,84]. In the gastrointestinal tract, mesenteric lymphatics regulate dietary lipids absorption, as well as the removal of inflammatory mediators generated during enteric immune activation [93]. Altered lymphatic contractility or excessive structural remodelling may impair immune cells egress, thereby sustaining local inflammatory loops that may contribute to visceral hypersensitivity. In musculoskeletal and periarticular tissues, impaired lymph drainage can favour fluid accumulation and alter the mechanical microenvironment, indirectly affecting both immune cell distribution and nociceptive signalling, with potential secondary consequences for tissue biomechanics and movement-related pain [11,78]. In severe pathological conditions, such as in the tumour microenvironment, neuropeptide-driven lymphangiogenesis and lymphatic remodelling acquire an additional immunological dimension. Expanding lymphatic networks can facilitate tumour antigen drainage and immune cell egress towards draining lymph nodes, potentially supporting antigen presentation and anti-tumour surveillance. However, the same lymphatic routes may also promote immune escape by shaping tolerogenic lymph node environments, supporting regulatory immune programmes, and allowing dissemination of tumour-derived signals and cells [94]. In this setting, lymphangiogenesis should therefore be viewed as a bidirectional modulator of immunity, capable of amplifying protective immune activation in some contexts while favouring immune evasion and chronic inflammation in others.

**Figure 5:**
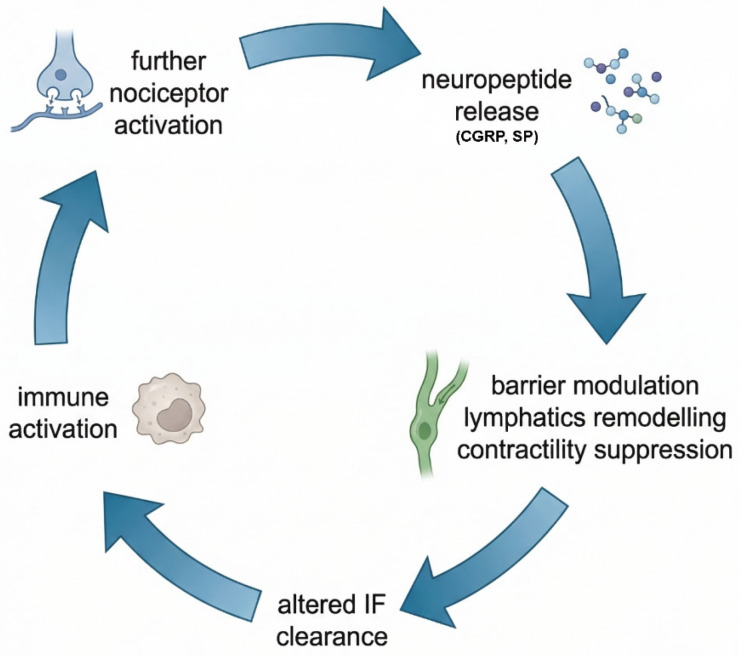
Neuro-lymphatic feedback loop linking nociceptive activation, lymphatic dysfunction and immune responses. Lymphatic vessels operate at the interface between nociceptive afferents and immune cell networks, dynamically regulating interstitial fluid clearance, inflammatory mediator distribution and immune cell trafficking. Prolonged nociceptor activation induces the release of neuropeptides, including CGRP and SP, which act on lymphatic endothelial and muscle compartments to modulate barrier properties, structural organisation and contractility. These coordinated effects impair lymphatic transport efficiency, leading to reduced interstitial fluid clearance and prolonged retention of cytokines, chemokines and other bioactive mediators within the interstitial space. The resulting accumulation of inflammatory signals promotes immune cell activation and sustains local inflammatory responses. In turn, increased concentrations of inflammatory mediators lower nociceptor activation thresholds and enhance peripheral sensitisation, driving further neuropeptide release. This establishes a self-amplifying neuroimmune feedback loop in which lymphatic dysfunction contributes to the persistence of inflammation and pain-related signalling.

Across different body compartments, a common integrative principle emerges: lymphatic vessels can finely regulate the spatial confinement versus dissemination [78] of inflammatory signals. Although direct experimental support remains uneven across tissues, available evidence converges on the concept that lymphatic function contributes to inflammatory signal handling by regulating mediator clearance and the spatio-temporal organisation of the interstitial microenvironment. Efficient lymphatic clearance promotes resolution by exporting cytokines, antigens and activated immune cells to draining lymph nodes, where adaptive responses are organised and inflammatory feedback loops may be modulated. In contrast, reduced lymph propulsion, barrier tightening that limits interstitial fluid entry, or disorganised hyperplastic networks with impaired functional coordination may increase the residence time of inflammatory mediators within the interstitium [78]. Prolonged exposure of nociceptors to such mediators may reinforce peripheral sensitisation and sustain neuropeptide release itself, thereby establishing a self-amplifying neuroimmune circuit, as illustrated in Figure 5.

Within this loop, lymphatic vessels function as modulators of signal persistence rather than as primary initiators of pain. Nevertheless, by determining clearance efficiency and immune trafficking patterns, they critically influence whether inflammatory responses remain transient or may evolve into chronic pain-associated states.

The consequences of these interactions remain context-dependent. During acute injury, transient modulation of contractility or adaptive lymphangiogenesis may enhance fluid removal and support immune resolution [78,84]. In this phase, lymphatic adjustments can be viewed as compensatory mechanisms that restore tissue homeostasis. However, when nociceptive activation becomes persistent, prolonged neuropeptide signalling may stabilise structural and functional alterations of the lymphatic network. Chronic reduction of propulsion, excessive or disorganised lymphangiogenesis, or sustained changes in endothelial barrier properties can collectively impair interstitial fluid drainage despite increased vessel density. Such maladaptive remodelling may entrench inflammatory niches and facilitate long-term immune activation (Figure 5). Thus, neuroimmune coupling in pain-related diseases cannot be fully understood without considering lymphatic physiology [4]. Lymphatic vessels integrate neural inputs, immune activity and mechanical forces within the tissue microenvironment. By controlling the kinetics and distribution of inflammatory mediators, they contribute to the transition from acute adaptive responses to sustained inflammatory or chronic pain states. In this perspective, lymphatics represent a regulatory intermediate through which nociceptive signalling and immune dynamics converge across both central and peripheral compartments [4].

## 5. Endogenous and Pain-Related Pharmacological Modulation of Lymphatic Function

### 5.1. Endogenous Opioid Signalling in Lymphatic Function and Adaptation

**Figure 6:**
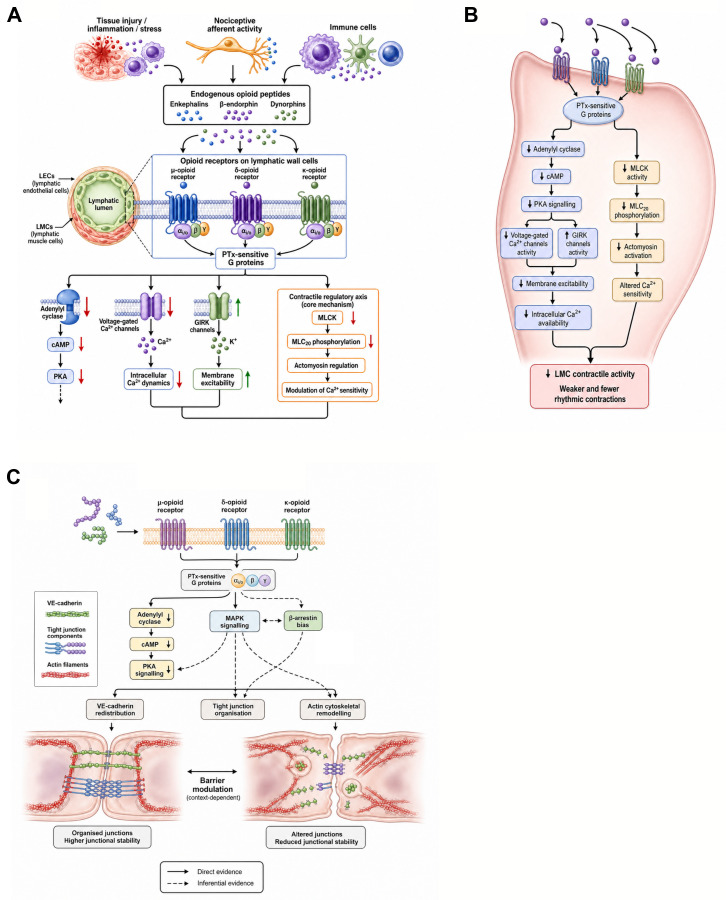
Endogenous and pharmacological opioid modulation of lymphatic function. (**A**). Core opioid signalling mechanism. Endogenous opioid peptides, including enkephalins, β-endorphin and dynorphins, are released during tissue injury, inflammation and stress and act through μ-, δ- and κ-opioid receptors expressed in peripheral tissues, including components of the lymphatic wall. These G protein-coupled receptors predominantly engage pertussis toxin-sensitive G proteins, leading to inhibition of adenylyl cyclase, reduced cAMP/PKA signalling, modulation of voltage-gated Ca^2+^ channels and activation of GIRK channels. Together, these pathways alter membrane excitability, intracellular Ca^2+^ handling and downstream contractile signalling. (**B**). Effects on lymphatic muscle cells. In LMCs, opioid receptor activation may reduce spontaneous lymphatic contractility by decreasing Ca^2+^ availability, limiting MLCK-dependent MLC20 phosphorylation and reducing actomyosin activation. Additional receptor subtype- and context-dependent mechanisms, including possible RhoA/ROCK modulation and β-arrestin-dependent signalling bias, may further fine-tune Ca^2+^ sensitivity and contractile tone. The resulting effect is a reduction or recalibration of contraction frequency and lymph propulsion. (**C**). Potential effects on lymphatic endothelial barrier function. Opioid-dependent signalling may also influence LEC behaviour by modulating MAPK-dependent pathways, cytoskeletal organisation and junctional protein dynamics, including VE-cadherin and tight junction components. These putative endothelial effects could alter barrier properties, interstitial fluid uptake and inflammatory mediator entry into lymphatic capillaries, thereby influencing clearance kinetics. Direct evidence for opioid-dependent regulation of LEC barrier function remains limited, and these mechanisms require validation in pain-relevant lymphatic settings.

Endogenous opioid peptides, including enkephalins, β-endorphin and dynorphins, are released in response to tissue injury, inflammation and stress, and constitute a central component of intrinsic analgesic systems [95]. Beyond their well-established effects on neuronal excitability, opioid receptors are expressed in peripheral tissues, including immune cells, vascular smooth muscle and, increasingly recognised, components of the lymphatic vasculature [96]. This distribution suggests that endogenous opioid signalling may modulate lymphatic physiology at multiple levels, integrating pain control with regulation of interstitial fluid handling and immune trafficking (Table 1). Opioid receptors (μ-, δ- and κ-subtypes) are G protein coupled receptors that predominantly couple to pertussis toxin-sensitive G proteins. Receptor activation inhibits adenylyl cyclase, reduces intracellular cAMP levels and attenuates PKA signalling. In addition, opioid receptor engagement modulates ion channel activity by inhibiting voltage-dependent Ca^2+^ channels and activating G protein-gated inwardly rectifying K+ (GIRK) channels, thereby influencing membrane excitability and intracellular Ca^2+^ dynamics [97] (Figure 6A). In smooth muscle cells, these signalling pathways can alter contractile behaviour through changes in Ca^2+^ availability, as well as Ca^2+^ sensitivity of the actomyosin apparatus. In collecting lymphatics, accumulating observations indicate that opioid receptor activation can modulate spontaneous contractions of lymphatic muscle. Opioid receptors have been detected in LMCs [98,99], and functional studies in isolated vessel preparations suggest that receptor activation may reduce contraction frequency and lymph propulsion [98]. At present, the most direct evidence in lymphatic vessels concerns opioid-dependent modulation of contractile behaviour, whereas several downstream signalling mechanisms remain inferred from broader smooth muscle physiology. Suppression of cAMP/PKA signalling can alter phosphorylation of key regulatory proteins involved in contraction, including MLCK (myosin light chain kinase), thereby reducing phosphorylation of MLC20 and dampening contractile force generation [88]. Concurrent inhibition of voltage-gated Ca^2+^ channels further limits Ca^2+^ influx during depolarisation, contributing to decreased LMC excitability (Figure 6B). In this context, endogenous opioids may act as physiological modulators that transiently restrain lymph propulsion during acute stress or inflammation. However, the effects of opioid signalling on lymphatic contractility are likely to be receptor subtype- and context-dependent. In smooth muscle cells, Ca^2+^ sensitivity is regulated by RhoA/ROCK-dependent pathways that act independently of cytosolic Ca^2+^ concentration [100]. Although opioid signalling has been shown to interact with RhoA-related pathways in some cellular contexts, direct evidence linking opioid receptor activation to RhoA/ROCK signalling in contractile cells remains limited. However, similar mechanisms in LMCs could fine-tune contractile tone without fully suppressing phasic activity. The balance between G-mediated inhibition of cAMP production, modulation of ion channels and potential β-arrestin-dependent signalling bias may determine whether endogenous opioid release results in net suppression or subtle recalibration of lymph propulsion.

In vascular endothelial cells, opioid receptor activation engages MAPK-dependent signalling pathways and modulates endothelial growth and survival responses [101]. Extrapolating to LECs, such signalling could potentially influence the distribution and stability of junctional proteins, including VE-cadherin and tight junction components, thereby modulating barrier properties and interstitial fluid uptake (Figure 6C). Direct experimental evidence in LECs remains limited, but the known intracellular targets of opioid receptors provide a plausible framework through which endogenous peptides could alter endothelial permeability and fluid handling. Accordingly, although opioid-dependent modulation of endothelial signalling is biologically plausible, the proposed effects on LEC barrier properties, junctional organisation and fluid uptake inferred from vascular endothelial data, should be interpreted with caution until directly validated in pain-relevant lymphatic settings.

Opioid signalling may also regulate lymphatic adaptation indirectly through immunomodulatory effects. Immune cells express all major opioid receptor subtypes [95] and are capable of both producing and responding to endogenous opioid peptides [102,103]. Activation of these receptors modulates cytokine production, NF-κB activity and MAPK signalling [102,104], thereby influencing the inflammatory microenvironment. Changes in cytokine profiles and macrophage phenotype are closely associated with altered production of lymphangiogenic growth factors such as VEGF-C and VEGF-D [82,105]. These factors in turn regulate VEGFR3-dependent signalling in LECs, which represents the canonical pathway driving lymphatic growth and structural remodelling [16,78,81]. Inflammatory mediators derived from activated immune cells, including macrophage-derived VEGF-C and VEGF-D, provide a key mechanistic link between immune activation and lymphatic expansion [77]. In chronic inflammatory states, sustained opioid receptor engagement may contribute to persistent alterations in the inflammatory microenvironment, including cytokine production and angiogenic signalling [106,107]. Through these indirect pathways, opioid signalling could therefore influence LECs and LMCs phenotype, extracellular matrix composition and valve competence, even in the absence of direct endothelial proliferation.

From a functional perspective, endogenous opioid signalling may represent a homeostatic mechanism linking nociceptive activation to controlled modulation of lymphatic function. During acute inflammation, transient opioid release may dampen excessive contractile activity, limit abrupt changes in interstitial fluid distribution and modulate immune cell trafficking in a manner that prevents overactivation of inflammatory circuits. Conversely, prolonged or dysregulated opioid signalling, whether due to persistent inflammation or repeated nociceptive stimulation, may contribute to reduced lymph propulsion, altered barrier regulation and maladaptive structural adaptation [99,108]. The distribution of opioid receptor subtypes across LMCs, LECs and perilymphatic immune cells is likely to vary between tissues, suggesting a regional heterogeneity in opioid-lymphatic interactions. A comprehensive characterisation of receptor subtype expression, signalling bias and downstream kinase activation profiles within lymphatic compartments remains an essential area for future investigation. Such in-depth studies will be necessary to determine whether endogenous opioid pathways primarily serve protective, adaptive roles in maintaining interstitial fluid homeostasis and immune balance, or whether they participate in the pathogenesis of lymphatic dysfunction associated with chronic inflammatory-related pain. In this framework, endogenous opioid peptides should be considered potential modulators of lymphatic function, as they provide an additional axis through which nociceptive activity can influence lymph propulsion, interstitial fluid clearance and organisation of inflammatory responses within tissues. Exogenous opioid analgesics act on the same receptor subtypes and intracellular signalling pathways described above for endogenous opioids. For this reason, their impact on LECs and LMCs is unlikely to differ qualitatively from endogenous ligands. Rather, the principal distinction resides in exposure profile, concentration and duration of receptor activation, which may amplify or prolong modulatory effects on lymphatic tone and barrier behaviour; pharmacological dosage should therefore be considered a quantitative extension of endogenous regulatory signalling.

### 5.2. Analgesics and Anaesthetics as Modulators of Lymphatic Physiology

Beyond endogenous regulatory systems, pain-related pharmacological agents may directly or indirectly influence lymphatic physiology. Local anaesthetics and general anaesthetic agents, widely employed in perioperative and interventional settings, possess molecular targets that overlap with key determinants of LECs and LMCs function. Although their primary clinical indication is the suppression of neuronal excitability, their action is not restricted to nociceptive fibres and may extend to vascular and lymphatic compartments, as experimental evidence indicates that local anaesthetics can directly modulate lymphatic contractile activity [109]. In addition, their well-established relaxant effects on smooth muscle suggest a broader impact on contractile tissues, including LMCs [110,111].

Local anaesthetics, such as lidocaine and bupivacaine, exert their principal effect through blockade of voltage-gated Na^+^ channels, thereby preventing action potential initiation and propagation in nociceptive afferents. However, voltage-dependent Na^+^ and Ca^2+^ channels are also expressed in vascular smooth muscle and, to a lesser extent, in LMCs. In collecting lymphatics, rhythmic contractions depend on coordinated depolarisation events, and pharmacological inhibition of these channels may therefore attenuate LMC excitability, reduce contraction frequency and diminish lymph propulsion. Experimental observations in isolated vessel preparations indicate that local anaesthetics can suppress spontaneous lymphatic contractions in a dose-dependent manner, an effect that appears reversible upon washout [109,112,113]. This effect is physiologically relevant because rhythmic lymphatic contractions contribute to the active redistribution of perioperative fluids and to the clearance of inflammatory mediators from the interstitium. Consequently, transient suppression of lymphatic contractility by local anaesthetics may reduce lymph propulsion, favour local fluid retention and delay the removal of pro-inflammatory signals in tissues exposed to high local drug concentrations. At present, the most direct evidence on lymphatic physiology concerns reversible suppression of spontaneous contractility, whereas endothelial-related signalling, including barrier regulation, remains less directly defined in pain-relevant lymphatic settings. Indeed, in addition to effects on LMCs, local anaesthetics may also influence endothelial function. In vascular cells, these agents have been shown to modulate intracellular Ca^2+^ dynamics, alter NO production and interfere with cytoskeletal organisation [114,115]. Whether similar mechanisms operate in LECs remains incompletely defined. Extrapolation from vascular endothelium suggests that such signalling could modify endothelial barrier properties and interstitial fluid uptake. Alterations in junctional protein distribution or endothelial-derived NO release might secondarily affect both capillary entry and collecting vessel contractility. Although direct studies on LECs remain limited, the known molecular targets of local anaesthetics suggest that transient changes in lymphatic barrier behaviour and propulsion may plausibly occur in tissues exposed to elevated local drug concentrations.

General anaesthetic agents introduce additional layers of modulation. Volatile anaesthetics and intravenous agents, such as isoflurane and propofol, primarily enhance inhibitory neurotransmission or depress central neuronal activity. However, they also exert systemic vascular effects. In vascular smooth muscle, several anaesthetic agents can induce hyperpolarisation and attenuate vascular tone regulation [116]. If comparable electrophysiological mechanisms are present in LMCs, they would be expected to reduce contraction frequency and tone, thereby transiently suppressing lymph propulsion during anaesthesia [111,112]. Moreover, anaesthetic-induced alterations in systemic haemodynamics, including reductions in arterial pressure and changes in vasomotion, may also indirectly influence extrinsic forces acting on lymphatic vessels, further modifying lymph transport dynamics.

The perioperative context is of particular relevance. Surgical trauma induces robust neuroimmune activation and inflammatory mediator release, conditions in which lymphatic function is critical for interstitial fluid clearance and immune cell trafficking. Concomitant exposure to anaesthetic agents may transiently dampen intrinsic contractility or modify barrier properties, thereby altering the kinetics of fluid redistribution and inflammatory resolution. Although such effects are likely reversible in most cases, repeated or prolonged exposure could influence postoperative course and recovery trajectories. In this perioperative setting, current literature indicates that anaesthesia-induced lymphatic dysfunction may contribute to altered interstitial fluid distribution and may therefore help explain part of the oedema burden observed after prolonged surgery or intensive care support [117]. Moreover, the magnitude and direction of pharmacological effects on lymphatic physiology are expected to be dependent upon the surrounding tissue context. Drug concentration, route of administration, tissue vascularisation and pre-existing inflammatory states may all contribute to determine whether lymphatic modulation is minimal or functionally significant. Unlike endogenous regulatory systems, which operate within tightly controlled physiological ranges, exogenous anaesthetic agents may transiently exceed these boundaries, leading to broader suppression of excitability across multiple cell types within the lymphatic wall.

In this framework, pain-related anaesthetic agents should be considered potential transient regulators of lymphatic function (Table 1), in addition to their neuronal effects [111]. By influencing LMC excitability, endothelial signalling and extrinsic mechanical drivers of lymph flow, these drugs may alter interstitial fluid handling and immune cell trafficking during periods of acute tissue stress. Recognition of these interactions broadens the physiological interpretation of perioperative fluid dynamics and inflammatory resolution in both acute and chronic pain contexts.

## 6. Therapeutic Perspectives on Lymphatic Regulation in Inflammatory Pain

The recognition of lymphatic vessels as active regulators of inflammatory signal persistence rather than passive conduits of fluid transport introduces a conceptual shift with potential therapeutic implications. Lymphatic barrier regulation, structural remodelling and intrinsic contractility have emerged as three interconnected levels through which nociceptive activity can influence immune dynamics and tissue fluid homeostasis [12,16,78,85]. From a translational perspective, these regulatory nodes should not be viewed as isolated molecular targets, but rather as components of an integrated physiological system whose coordinated function determines whether inflammatory responses resolve or persist.

Current pain therapies primarily aim to suppress neuronal excitability or dampen immune activation. Beyond opioid-based approaches, clinically used pharmacological strategies for inflammatory pain include non-steroidal anti-inflammatory drugs (NSAIDs), glucocorticoids, local anaesthetics and, in selected immune-mediated inflammatory disorders, cytokine-directed biological agents. NSAIDs primarily act by inhibiting cyclooxygenase-dependent prostaglandin synthesis, thereby reducing inflammatory mediator production and peripheral nociceptor sensitisation [118,119]. However, repeated or prolonged NSAID use is limited by well-recognised gastrointestinal, renal and cardiovascular adverse effects [120,121]. Glucocorticoids exert broader anti-inflammatory and immunosuppressive effects by regulating inflammatory transcriptional programmes, including pathways controlling cytokine and chemokine expression [122]. Their long-term use, however, is constrained by systemic adverse effects involving metabolic, musculoskeletal, cardiovascular, ocular and immune functions [123]. In chronic immune-mediated inflammatory diseases, biological therapies targeting cytokine pathways such as TNF-α, IL-1, IL-6 or IL-17 can reduce inflammatory activity and pain-related disease burden, but their use is limited by cost, patient heterogeneity and risks related to systemic immune suppression [124]. Finally, CGRP-pathway therapies, including monoclonal antibodies and Gepants, represent a clinically established neuropeptide-directed strategy in migraine, but the consequences of sustained CGRP modulation for lymphatic endothelial regulation and fluid clearance remain insufficiently characterised [12,19,125]. However, inflammatory signal persistence within tissues is not solely a function of mediator production, as it is also critically dependent on clearance efficiency and spatial redistribution [78]. In this context, therapeutic strategies that modulate neuropeptide signalling, vascular tone or immune activation may exert previously underappreciated effects on lymphatic function (Table 1). Rather than proposing lymphatics as primary analgesic targets, they may be more appropriately considered as regulatory amplifiers of local inflammatory microenvironments. Interventions that inadvertently impair lymph propulsion, destabilise barrier properties or promote maladaptive remodelling could prolong inflammatory niches despite effective neuronal suppression [16,78]. Conversely, strategies that preserve or restore coordinated lymphatic function may facilitate resolution by improving interstitial fluid clearance and immune cell trafficking.

A translationally relevant aspect concerns the neuropeptide-lymphatic interface. Pharmacological modulation of CGRP or SP signalling has already entered clinical practice in specific pain conditions, most notably migraine [12]. While these interventions were developed to reduce neuronal sensitisation, their systemic impact on lymphatic regulation has not been systematically examined. Given that neuropeptide signalling influences barrier behaviour, lymphangiogenic balance and contractile coordination [19,67,85], future therapeutic frameworks may benefit from assessing lymphatic outcomes alongside classical pain endpoints (Table 1). Such evaluation would not imply direct targeting of lymphatics, but rather recognition that neuroimmune therapies operate within a broader tissue-regulatory network. Another therapeutic consideration relates to the restoration of proper lymph propulsion. In chronic inflammatory contexts, structural expansion of lymphatic networks does not necessarily equate to functional efficiency. Disruption of rhythmic contractions, impaired valve function or altered endothelial-muscle coupling may compromise effective lymph transport, even in the presence of increased vessel density [78,126]. From a physiological standpoint, resolution of inflammation may require not only suppression of pro-inflammatory mediators but also the re-establishment of proper lymph propulsion and controlled barrier dynamics [78,84]. Interventions aimed at preserving contractile responsiveness or preventing sustained suppression of lymph propulsion could therefore complement anti-inflammatory strategies. Such approaches would focus on restoring physiological coordination rather than globally enhancing lymph flow, which could carry unintended immunological consequences. The concept of “resolution-supportive lymphatic physiology” may provide a useful translational framework, in which therapeutic success is not defined solely by reduced mediator production, but also by restoration of effective signal export from the interstitium to draining lymph nodes. Efficient clearance limits prolonged nociceptor exposure to inflammatory mediators and reduces the likelihood of self-amplifying neuroimmune loops. At the same time, excessive or disorganised lymphangiogenesis may reinforce chronic immune activation by sustaining antigen presentation and immune cell trafficking. The transition between adaptive and maladaptive lymphangiogenesis may depend on the duration, intensity and cellular context of neuropeptide signalling. Transient activation of SP–NK1R signalling can support organised VEGFR3-dependent lymphatic sprouting and thereby favour mediator export during tissue repair. In contrast, sustained nociceptive neuropeptide release, together with persistent immune-cell activation, may maintain elevated VEGF-C and VEGF-D availability and promote excessive or poorly coordinated lymphatic expansion. Under these conditions, newly formed vessels may increase network density without proportionally improving transport efficiency, particularly if structural remodelling is accompanied by altered barrier organisation, impaired valve patterning or reduced contractile coordination. Thus, the adaptive-to-maladaptive “switch” is therefore best understood as a context-dependent transition rather than as a single molecular event. Therapeutic modulation must therefore aim to maintain a functional balance, promoting coordinated clearance while avoiding structural changes that stabilise inflammatory niches [78,127].

The perioperative and interventional settings represent additional contexts in which lymphatic physiology may influence recovery course. Surgical trauma, anaesthetic exposure and acute inflammatory activation converge during a period in which fluid redistribution and immune cell trafficking are dynamically regulated [78,84]. Although most pharmacological effects on lymphatic behaviour are likely transient, repeated or prolonged perturbations could influence oedema formation, tissue repair and immune surveillance [77,78]. Incorporating lymphatic function into perioperative research paradigms may refine understanding of postoperative inflammation and recovery without need of direct lymphatic-targeted interventions.

Despite these emerging conceptual links, significant knowledge gaps remain. An additional limitation is the difficulty of isolating lymphatic-specific effects from the closely interconnected vascular, immune and neuronal processes operating within the same inflammatory microenvironment, including changes in vascular permeability, immune activation and nociceptor sensitisation. Interspecies variability in lymphatic vessel structure, perilymphatic innervation and neuropeptide responsiveness may further limit the generalisability of mechanistic findings derived from animal models or tissue-specific preparations to broader pain-relevant conditions. Moreover, comprehensive mapping of opioid, CGRP and NK1R receptor distribution across LECs, LMCs and perilymphatic immune populations in different tissues is still lacking. Comparative analyses between central and peripheral lymphatic beds are limited, and the temporal dynamics of lymphatic adaptation during acute-to-chronic transitions remain insufficiently characterised. Quantitative models linking mediator production, lymphatic clearance efficiency and nociceptor sensitisation thresholds remain largely unavailable, limiting a more precise definition of how lymphatic dysfunction contributes to inflammatory signal persistence across tissues. Furthermore, most mechanistic insights derive from isolated vessel preparations or specific disease models [12,69], underscoring the need for integrative approaches that combine functional imaging, molecular profiling and longitudinal inflammatory assessment. Development of experimental platforms capable of simultaneously quantifying lymph flow, barrier behaviour and immune trafficking would substantially advance the field.

From a broader perspective, incorporating lymphatic physiology into models of inflammatory pain encourages a shift from a neuron-centric to a systems-level understanding of neuroimmune coupling. Lymphatic vessels integrate neural signals, immune activity and mechanical forces within the tissue microenvironment [11,78]. By regulating the kinetics and spatial distribution of inflammatory mediators, they can shape the duration and amplification of nociceptive signalling [78,84]. Future translational research should therefore consider lymphatic function not as a peripheral adjunct to inflammation, but as a modifiable determinant of inflammatory signal dynamics [4]. In this context, therapeutic strategies for pain-related inflammatory conditions may be strengthened by recognising the lymphatic system as an intermediate regulatory interface linking neural activation to immune resolution. While direct lymphatic targeting remains premature, preserving coordinated barrier function, structural organisation and intrinsic contractility may prove essential for effective resolution of inflammatory microenvironments [16,67,85]. Continued investigation at the intersection of neurobiology, immunology and lymphatic physiology will be required to define how modulation of this axis can be safely and effectively integrated into future therapeutic paradigms.

## 7. Conclusions: Integrative Framework and Open Questions

The lymphatic system has traditionally been regarded as a network of passive conduits responsible for fluid return and immune cell transport. However, an alternative conceptual framework suggests that lymphatics function as dynamic regulators of inflammatory signal persistence. By integrating barrier behaviour, structural plasticity and intrinsic contractility, they influence not only the magnitude of tissue inflammation but also its spatial confinement and temporal evolution. In inflammatory pain states, this regulatory involvement becomes particularly relevant, as the duration and distribution of inflammatory mediators critically shape nociceptor sensitisation and the transition from acute to chronic pain-related conditions. Rather than acting as primary drivers of inflammation, lymphatic vessels appear to modulate the efficiency with which inflammatory signals are cleared, redistributed or retained within tissues, thereby shaping their persistence within the interstitium. Neural activation, immune responses and intrinsic and extrinsic mechanical forces converge on the lymphatic wall, where endothelial and muscle components contribute to coordinated changes in permeability, network architecture and propulsion. Through this integrative role, lymphatics contribute to the kinetics of inflammatory resolution. Efficient local clearance may limit prolonged nociceptor exposure to cytokines and growth factors, whereas impaired lymph propulsion or maladaptive remodelling may stabilise inflammatory niches and sustain neuroimmune amplification loops. The balance between these outcomes is likely determined by tissue-specific organisation, receptor distribution and the temporal pattern of neural activation. However, the strength of available evidence remains uneven across tissues and experimental conditions, and some components of this framework still rely on indirect mechanistic inference rather than direct demonstration in pain-relevant settings.

This perspective encourages a shift from a neuron-centric interpretation of inflammatory pain toward a systems-level view in which clearance dynamics represent an additional regulatory variable. Signal persistence within the interstitium is not solely dictated by mediator production, but also by the capacity of the tissue to export those signals in a coordinated manner. Lymphatic physiology becomes an intermediate layer linking nociceptive activity to immune resolution. This does not reposition lymphatics as isolated therapeutic targets. Instead, it situates them within a broader regulatory network whose coordinated function may influence disease course. Important questions remain to be addressed, regarding the quantitative contribution of lymphatic transport to inflammatory signal kinetics, the heterogeneity of lymphatic regulation across central and peripheral compartments, and the temporal dynamics governing the shift from adaptive modulation to maladaptive persistence. At present, the quantitative contribution of lymphatic transport to inflammatory signal persistence across tissues remains insufficiently defined, and causal relationships have been established only in selected experimental settings. Addressing these questions will require integrative approaches capable of simultaneously assessing neural activation, immune mediator distribution and lymphatic propulsion in vivo. Advances in imaging, molecular profiling and functional analysis of lymph flow will be essential to clarify how this axis operates across different tissues and pathological contexts. Translational progress will therefore require experimental and clinical approaches that integrate lymphatic, neural and immune readouts within the same framework, together with standardised in vivo endpoints enabling comparison across models and disease settings. Collectively, these considerations support the view that lymphatics serve as integral components of neuroimmune integration, shaping the spatial and temporal landscape of inflammatory pain. Recognising this regulatory dimension refines our understanding of how inflammatory signals are sustained or resolved and establishes a physiologically grounded framework for future investigation of pain-related disorders.

**Table 1:** Comparison of mediator effects.

Mediator	Lymphatic Target	Tissue/ Model	Type of Evidence	Functional Consequence	Relevance to Pain	Strength of Evidence	Key References
**CGRP**	Endothelial Barrier (LECs)	Meningeal (In vivo, MRI, in vitro)	Direct	VE-cadherin redistribution; junction tightening	Impaired CSF clearance; migraine sensitization	High (Meningeal)	[12,13,14,20,67,68,69]
	Contractility (LMCs)	Mesenteric/Dermal (Isolated)	Direct	NO-dependent inhibition of pumping frequency	Interstitial fluid retention and mediator stasis	Moderate (Peripheral)	[84,85,86,87,88,89]
**Substance P**	Lymphangiogenesis	Dermal/Macrophages (In vitro)	Direct	VEGFR3 upregulation; vessel sprouting	Adaptive resolution vs. maladaptive persistence	High	[70,71,72,76,77,78,79,80]
	Contractility (LMCs)	Isolated collecting vessels	Direct	Increased contraction frequency and amplitude	Facilitation of mediator export/clearance	Moderate	[70,71,72,73,74,75,84,85,86]
**Endogenous Opioids**	Contractility (LMCs)	Isolated vessels/Smooth muscle	Direct/Indirect	Reduced cAMP; suppression of pump frequency	Potential prolongation of inflammatory niches	Moderate/Hypothesis	[84,85,86,99,100,101,102,103,104]
**Local Anaesthetics**	LMC Excitability	Isolated vessel preparations	Direct/Indirect	Blockade of Na^+^/Ca^2+^ channels; pump suppression	Transient stasis; delayed oedema resolution	Moderate/Hypothesis	[108,109,110,111,112,114,115]
**General Anaesthetics**	LMC Membrane Potential and Tone	Systemic/Surgical models	Direct/Indirect	Hyperpolarization and Reduced vessel tone and systemic lymph transport	Delayed resolution of trauma-induced inflammation	Moderate/Hypothesis	[84,85,86,116,126]
**Anti-CGRP (mAbs/gepants)**	CLR/RAMP1 Receptor Complex	Meningeal/Systemic (Clinical & Models	Indirect /Clinical	Blockade of CGRP-mediated barrier tightening and contractile inhibition	Restoration of clearance kinetics Unknown systemic impact on peripheral clearance	High (Meningeal) Moderate (Periphery)	[12,13,14,19,66]

## Data Availability

No new data were collected for this review report.

## Abbreviations

The following abbreviations are used in this manuscript:

CGRP	Calcitonin Gene-Related Peptide
SP	Substance P
LECs	Lymphatic Endothelial Cells
LMCs	Lymphatic Muscle Cells
CLR	Calcitonin Receptor-Like Receptor
RAMP1	Receptor Activity-Modifying Protein 1
NK1R	Neurokinin-1 Receptor
VEGF-C/VEGF-D	Vascular Endothelial Growth Factor C/D
VEGFR3	Vascular Endothelial Growth Factor Receptor 3
CSF	Cerebrospinal Fluid
